# Idiopathic Omental Infarction, Diagnosed and Managed Laparoscopically: A Case Report

**DOI:** 10.1155/2013/193546

**Published:** 2013-08-26

**Authors:** Ahmed AbdulAziz, Tamer El Zalabany, Abdul Rahim Al Sayed, Ahmed Al Ansari

**Affiliations:** Department of General Surgery, Bahrain Defense Force Hospital, Off Waly Alahed Avenue, P.O. Box 28743, West Riffa, Bahrain

## Abstract

Idiopathic omental infarction is a rare cause of acute abdomen in adults, and the clinical finding can mimic acute appendicitis. Although idiopathic omental infarction is uncommon, the incidence of its detection has become more frequent as a result of advances in radiological technologies. We reported on a 21-year-old man who presented with sudden onset of intermittent right lower quadrant abdominal pain for seven days. The pain became more localized at the right iliac fossa (RIF) at day 2 before admission. A physical examination revealed a fever (38.2°C), severe RIF tenderness, mass-like fullness, and positive rebound tenderness. A CT of the abdomen showed inflammatory changes and increased fat density mass in the right upper quadrant measuring 5 × 4 cm representing focal panniculitis. However, the appendix was visualized normally and the findings were not in favor of acute appendicitis. Diagnosis was carried on laparoscopically. Serosanguinous free fluid was found in all abdominal quadrants. A 6 × 4 cm gangrenous omental mass was noted. The omental mass was excised and an appendectomy was performed. In summary, omental infarction should be considered as a deferential diagnosis for acute right-sided abdominal pain, especially if the clinical finding does not correspond to appendicitis.

## 1. Introduction

 Idiopathic omental infarction due to torsion of the omentum is a rare cause of acute abdomen. Torsion of the omentum is a condition in which the organ twists on its long axis to such an extent that its vascularity is compromised [1, 2]. Among a variety of acute abdomens, acute torsion of omentum is the least suspected under the impression of, most commonly, common cases such as acute appendicitis, acute cholecystitis, acute diverticulitis, mesenteric thrombosis, ovarian cyst, and perforated peptic ulcer [3]. Most patients present with acute right-lower quadrant pain, and not surprisingly, they are usually misdiagnosed as having appendicitis [2]. Ultrasonography (US) and computed tomography (CT) scanning are helpful tools for identifying the characteristic signs of omental infarction and signifying the deferential diagnosis. Laparoscopy is used in the diagnosis and treatment of such rare conditions.

## 2. Case Presentation 

**Figure 1:**
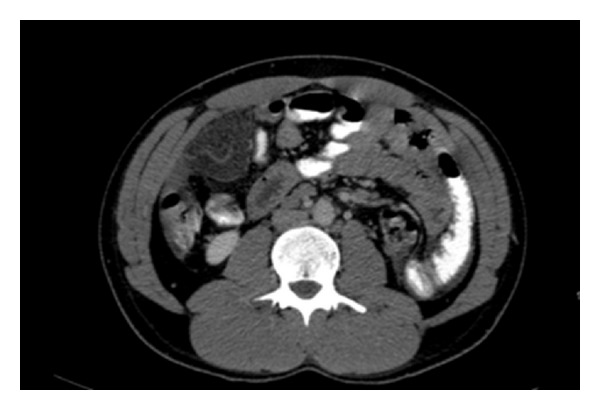
CT scan showing increased fat density mass in the upper quadrant.

 A 21-year-old male presented to the Emergency Department with a history of dull spasmodic abdominal pain for 7 days, and the pain became more localized at the right iliac fossa (RIF) at day 2, before admission. The pain increased in intensity throughout the day, was not relieved by any analgesia or antispasmodic medications, and was aggravated with movement, cough, and straining. The pain was associated with nausea, decreased appetite, and vomiting. There were no other associated symptoms such as change in bowel habit and urinary symptoms. There was no history of trauma or surgery. Neither obesity nor weight loss was noted. On examination, the patient was febrile with a temperature of 38.2°C, with stable vital signs. An abdominal examination revealed severe RIF tenderness, associated with guarding. Mass like fullness and positive rebound tenderness were observed in the RIF. The bowel sound was normal. His laboratory investigations showed a white cell count of 11,800 /mmc with 65.5% polymorphonuclear cells, HB: 15.2, and ESR 15 mm/hr. Amylase was within normal range. Abdominal plain film and erect chest radiographs showed no active disease. The urine analysis was normal. Abdominal ultrasonography showed a small amount of free fluid noted in the Morison's pouch infrahepatic without any other abnormalities. Computed tomography of the abdomen was performed and showed inflammatory changes and increased fat density mass in right upper-quadrant measuring 5 × 4 cm representing focal panniculitis (Figure 1). Free fluid was noticed in the infrahepatic and both paracolic gutters as well as around the appendix. 

**Figure 2:**
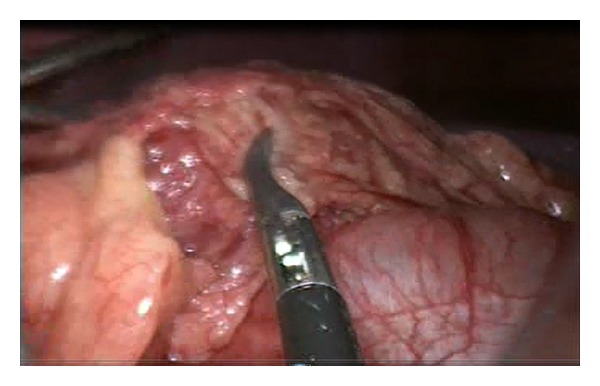
It shows torsion of omental masson laproscopic examination.

**Figure 3:**
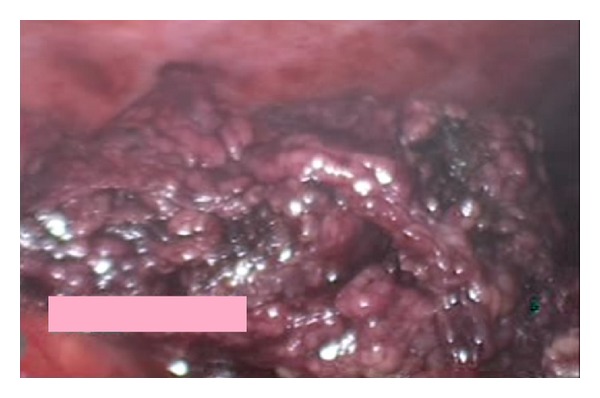
It shows a gangrenous omental mass on laparoscopic examination.

 However, the appendix was visualized normally and the findings were not in favor of acute appendicitis. The patient was admitted and kept NPO with intravenous fluid and analgesia. He was started on an antibiotic. Consent was given for diagnostic laparoscopy and an appendectomy under general anesthesia. During the diagnostic laparoscopic procedure, a serosanguinous free fluid was found in all abdominal quadrants. In addition, a 6 × 4 cm gangrenous omental mass was noted (Figures 2 and 3). The appendix was within normal limits. The omental mass was excised by appendectomy and samples of both specimens were sent out for a histopathology examination. The postoperative course was uneventful. The patient was doing well in the following days, started a normal diet, and was discharged with no complications. At a follow-up exam in the clinic, the histopathological report revealed congested omental tissue with focal necrosis and a mild fibroblastic reaction, confirming the diagnosis of omental torsion. The appendix showed no significant mural inflammation.

## 3. Discussion

 Torsion of the omentum is the main reason for omental infarction and it is classified as primary or secondary torsion. Primary torsion presents without any intra-abdominal pathogenic signs, and secondary torsion can present due to a secondary cause such as cysts, tumors, adhesions, or hernia. Primary omental torsion occurs idiopathically when a mobile segment of omentum rotates around a proximal fixed point in the absence of any associated intra-abdominal pathology. It can be predisposed by trauma, hyperperistalsis, and anatomical variations of the omentum itself, for example, accessory omentum, bifid omentum, irregular accumulations of omental fat in obese patients, and narrowed omentum pedicle. The higher incidence of torsion on the right side of the omentum is related to the greater length and mobility of that side, which leaves it more prone to twist itself along its long axis, leading to compromise the vascularity [1]. Secondary torsion is more common than primary torsion and is associated with abdominal pathology, including cysts, tumors, intra-abdominal inflammation, postsurgical scarring, and hernial sacs. Most cases of secondary torsion occur in patients with inguinal hernias [4].

 Most of the cases are presented as acute right-sided abdominal pain and misdiagnosed as acute appendicitis. Our patient complained of one week of dull abdominal pain, which became severe and localized at RIF, and the examination revealed RIF tenderness and the presence of a mass. The clinical picture of our patient with acute appendicitis shows mass formation. However, the US image was not informative as to the extent and our diagnosis required confirmation with an advanced radiological method such as CT abdomen. The increasing use of high-quality imaging, especially computerized tomography, in the diagnosis of appendicitis and the acute abdomen, has allowed preoperative diagnosis to be made much more often [5]. Although US findings are usually evaluated as normal [6], sometimes US may show a complex mass, a mixture of solid material, and hypoechoic zones. US is considered a diagnostic procedure useful for ruling out other acute abdominal conditions. However, CT has been shown as having a high sensitivity and specificity for the diagnosis of intraperitoneal focal fat infarction [7]. Preoperative US or CT scan is mandatory and the preoperative diagnosis can be accurately accomplished using these procedures. 

 The decision was made to use diagnostic laparoscopy followed by appendectomy. A laparoscopic approach enables the detection of other intra-abdominal masses and to identification of any associated pathology. In addition, a complete examination of the abdominal cavity to confirm the diagnosis, aspiration and washing of the peritoneum, and decreased postoperative pain and wound-related complications can be achieved with a laparoscopic approach. Although some cases require surgical intervention, nevertheless, surgical treatment of omental infarction seems to be limited to those with complications, such as failure of conservative management, omental abscess, bowel obstruction, and in cases of uncertain diagnosis. 

 Conservative treatment with bed rest and anti-inflammatory medications are advised before operating in cases in which the diagnosis is confirmed by US or CT and in hemodynamically stable patients [8]. In our case this was not applied. This approach has been associated with the development of omental abscesses in some patients [8]. Because of the severity of the presentation of our patient, surgical intervention was advised. Laparoscopic exploration should be considered as it can be both diagnostic and therapeutic and is associated with low morbidity [1, 9–11]. Laparoscopic resection of the involved omentum provides definitive treatment with a short hospitalization and rapid recovery. 

## 4. Conclusion

 Omental infarction should be considered with any patient presenting with acute right lower-quadrant pain. Abdominal ultrasonography and computed tomography should be used as initial diagnostic measures. If this fails, diagnosis and treatment of omental torsion can be achieved by laparoscopy with the advantage of determining the cause; decreased postoperative pain and wound-related complications; and short hospitalization and quick recovery. Further studies are required to compare the appropriateness between conservative and surgical management.
